# In Vitro Synergistic Effect of Colistin with Fosfomycin Against Carbapenem-Resistant Klebsiella pneumoniae

**DOI:** 10.7759/cureus.66295

**Published:** 2024-08-06

**Authors:** Chethankumar P, Tejashree A, Neetha S Murthy, Morubagal Raghavendra Rao

**Affiliations:** 1 Microbiology, JSS Medical College and Hospital, JSS Academy of Higher Education and Research (JSSAHER), Mysore, IND

**Keywords:** broth microdilution, multidrug-resistant organisms, fosfomycin, colistin, checkerboard method, carbapenem-resistant klebsiella pneumoniae, synergistic effect

## Abstract

Background: The dwindling antibiotic reserve owing to augmented drug-resistant bacteria is a major handicap for treating physicians. *Klebsiella pneumoniae*, a gram-negative encapsulated member of *the Enterobacteriaceae* family, is one such pathogenic bacteria. Carbapenemase-producing *Klebsiella pneumoniae is *globally recognized as one of the most critical bacterial threats to public health due to its extremely limited treatment options. Carbapenem-resistant *Klebsiella pneumoniae *(CRKP) infections pose therapeutic challenges due to simultaneous resistance to various other groups of antibiotics. In this study, we have evaluated the synergistic effect of fosfomycinagainst CRKP isolates when used in combination with colistin by applying the Checkerboard method.

Methods: A laboratory-based prospective study was conducted in the Department of Microbiology, JSS Hospital, Mysuru, for a period of one year after obtaining ethical clearance. *Klebsiella pneumoniae* isolates obtained from clinical samples were screened for carbapenem resistance by the VITEK-2 compact system (bioMérieux, Marcy-l'Étoile, France). The minimum inhibitory concentration (MIC) of colistin and fosfomycin was individually ascertained by broth microdilution (BMD). Finally, the synergistic activity of the fosfomycin-colistin combination was determined by the BMD-based Checkerboard method.

Results: Among the 50 CRKP isolates, 36 (72%) isolates showed synergism, eight (16%) isolates showed indifference and six (12%) isolates showed partial synergism, while none of them showed additivity and antagonism by the Checkerboard method. These results are found to be statistically significant (chi-square value of 116.204 and p-value of < 0.00001).

Conclusion: This study showed a promising in-vitro synergy between the drugs fosfomycin and colistin by Checkerboard BMD testing protocol. Colistin being a reserve antibiotic, monotherapy comes with the limitations of higher chances of resistance as well as toxicity, which can be overcome by combination therapy, thereby decreasing CRKP-associated mortality rates and delivering holistic patient benefit.

## Introduction

*Klebsiella pneumoniae* is a gram-negative bacteria belonging to the Enterobacteriaceae family [1] and is commonly associated with hospital-acquired infections like urinary tract infections, ventilator-associated pneumonia, hospital-acquired pneumonia, surgical site infections, intra-abdominal infections, bloodstream infections, and skin and soft tissue infections [2, 3]. *Klebsiella pneumoniae* produces carbapenem-hydrolyzing β-lactamases like class A Ambler (KPC type), class B Ambler (VIM, NDM, and IMP types), and class D Ambler (OXA-48), which seems primarily responsible for the organism's resistance to carbapenem [4], and loss of outer membrane porin proteins like OmpK35 and OmpK36 also contributes to carbapenem resistance in *Klebsiella pneumoniae* [5].

The rise of carbapenemase-producing *Klebsiella pneumoniae* has rapidly expanded over the past decade and become a major public health problem [6]. This accounts for the heightened morbidity and mortality associated with carbapenem-resistant *Klebsiella pneumoniae* (CRKP) infections [7]. The WHO has emphasized carbapenem-resistant Enterobacteriaceae (CRE), particularly CRKP, as one of the most prevalent immolating diseases [2]. Patients with CRE infections frequently have severe illnesses, which may contribute to the longer time to active therapy and pharmacologic restrictions on available treatment regimens [8].

Infections that are caused by CRKP are challenging and difficult to treat because of simultaneous resistance to various other groups of antibiotics, resulting in limited therapeutic options [9]. The advocation of single antibiotic therapy with colistin, fosfomycin, and tigecycline is inadequate in CRKP infections as it produces only modest clinical amelioration [10]. Monotherapy is associated with limitations such as the emergence of resistance, pharmacokinetic limitations, and toxicity during treatment; on the other hand, combination therapy overcomes these limitations and decreases the CRKP-associated mortality rate [11]. Given the scenario of the sparsity of novel antimicrobial agents, older antibiotic revaluation for advocacy of synergistic amalgamations presents an attractive option [12]. The use of the proper combination therapy is a crucial tactic for preventing the development of antibiotic-resistant bacteria [13].

The purpose of the combination therapy is to minimize toxicity and enhance the antibacterial efficacy of the drug. The in vivo effectiveness (synergistic, antagonistic, or additive) of the proposed combination therapy can be reasonably protracted based on the in vitro synergy testing methods like the E-strip method, the Checkerboard method, and the time-kill assay [14]. In view of the aforementioned scenario, the current study was undertaken with the aim of analyzing the in vitro additive, indifference, and synergistic effect by using the combination of colistin with fosfomycin against *Klebsiella pneumoniae* isolates resistant to carbapenems.

## Materials and methods

Study design and sample size calculation

A prospective laboratory-based study was conducted in the Department of Microbiology of JSS Hospital, a tertiary care hospital in Mysuru, South India, between January 2022 and February 2023, and institutional ethical clearance was obtained (approval number: JSS/MC/PG/31/2022-23). The sample size was calculated by using the following formula:

n = Z^2^ [p (1-p)] / d^2^,

where n = required sample size, Z = value for the confidence level (95% (1.96)), d = desired level of precision (13.9% (0.139)), and p = estimated prevalence (50% (0.5)). Thus, a total of 50 CRKP isolates were included in this study obtained by convenience sampling technique from different patient samples sent for routine culture, and susceptibility testing was studied for in vitro synergism between colistin and fosfomycin.

Isolation, identification, and antimicrobial susceptibility testing (AST) of suspect *Klebsiella *strains

The clinical samples reaching the microbiology laboratory were processed according to standard protocols. Samples were plated onto Blood agar (HiMedia, Thane (West), India) and MacConkey agar (HiMedia). Simultaneously, all the clinical specimens were also subjected to direct microscopic examination. Suspected *Klebsiella* colonies were identified based on their macroscopic colony morphology and microscopic appearance. Biochemical tests were done for species-level phenotypic identification of the *Klebsiella pneumoniae* isolates. The confirmed *Klebsiella pneumoniae *strains were processed in the VITEK-2 Compact system (bioMérieux, Marcy-l'Étoile, France) as per manufacturer instructions to detect the antibiotic susceptibility pattern by using VITEK-2 AST-N405 cards (bioMerieux). The carbapenem (meropenem, imipenem, and ertapenem) minimum inhibitory concentration (MIC) was determined by the VITEK-2 Compact system. Carbapenemase-producing CRKP isolates were confirmed by the Modified Hodge test. 

Fosfomycin and colistin MIC determination by broth microdilution (BMD) test

Fosfomycin MIC was determined by using Micropro BMD fosfomycin strips (256 µg/ml to 0.25 µg/ml) according to the manufacturer's instructions (Microxpress, Goa, India), and results were interpreted as MIC≤32-sensitive and MIC>32-resistant (The European Committee on Antimicrobial Susceptibility Testing (EUCAST) guidelines 2023). Colistin MIC was determined by using colistin powder (HiMedia) form as per the standard guidelines (EUCAST guidelines 2023 and Clinical & Laboratory Standards Institute (CLSI) guidelines 2022) and results were interpreted as MIC≤2-sensitive and MIC>2-resistant (EUCAST 2023).

Synergy testing

**Figure 1:**
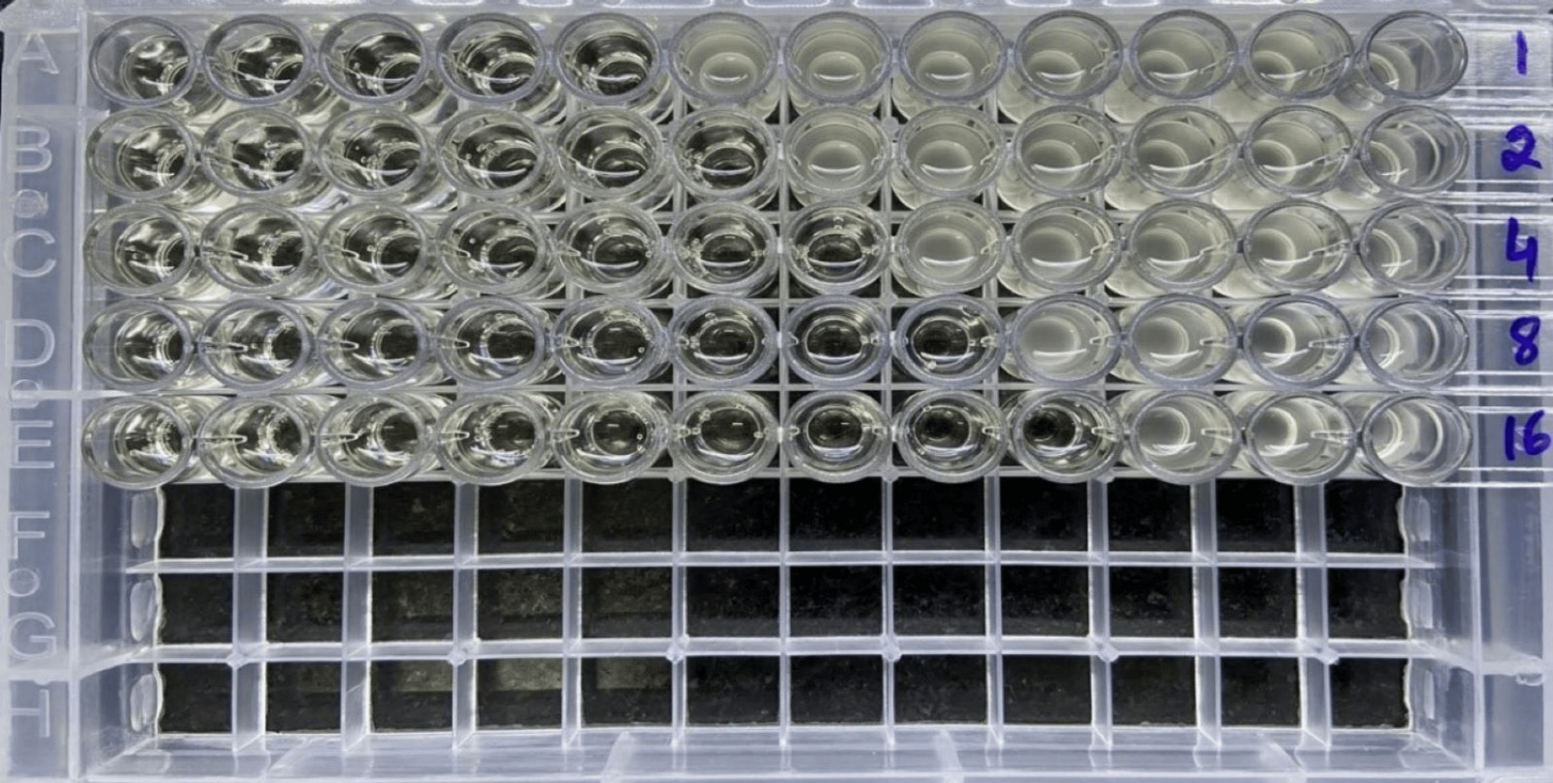
Isolate number 12: Synergistic activity of fosfomycin and colistin by broth microdilution-based checkerboard assay; Columns 1 to 11 contain fosfomycin in concentrations ranging from 256 µg/ml to 0.25 µg/ml and rows A to E contain colistin in concentrations ranging from 1 µg/ml to 16 µg/ml respectively.

The Checkerboard method was performed by using colistin (powder form) and Micropro BMD fosfomycin strips (256 µg/ml to 0.25 µg/ml). Fosfomycin and colistin MICs were predetermined and recorded by BMD. Two-fold dilution of fosfomycin pre-coated on BMD strips from column 1 (256 µg/ml) to column 11 (0.25 µg/ml) was used. Column 12 served as the control well. Two-fold dilution of colistin was carried out by taking concentrations less than, equal to, and greater than their MIC values determined previously [15]; 200 μl of the calculated colistin dilutions were dispersed to all wells in rows A to E [14]. Thus, a checkerboard consists of columns where wells contain the same amount of drug diluted along the x-axis and rows contain the same amount of drug diluted on the y-axis. Each well contains a unique combination of both drugs tested [16]. Inoculum (200 μl) of the CRKP isolate to be tested was added to all the wells such that each well houses a specific combination of the two antibiotics studied against the isolate along with growth control as depicted in Figure 1.

Incubation was performed at 37°C for 22-24 hours. The fractional inhibitory concentration (FIC) index for each drug was determined by using the lowest concentrations in both the column and row where no visual growth (turbidity) was observed throughout the entire row and column [17]. The FIC of colistin and fosfomycin was calculated by the following formula [18]: FIC of fosfomycin = MIC of fosfomycin in combination with colistin/MIC of fosfomycin alone, and FIC of colistin = MIC of colistin in combination with fosfomycin/MIC of colistin alone. Next, the mean fractional inhibition concentration index (FICI) was calculated using the formula: mean (FICI) = FIC of fosfomycin + FIC of colistin. The presence or absence of synergism was evaluated based on the FICI values (Table 1).

**Table 1:** Interpretation of synergism based on the fractional inhibitory concentration index (FICI)

Description	Fractional inhibitory concentration index (FICI)
Synergism	≤0.5
Partial synergism	0.5 to 1
Additivity	=1
Indifference	1 to 4
Antagonism	>4

## Results

**Figure 2:**
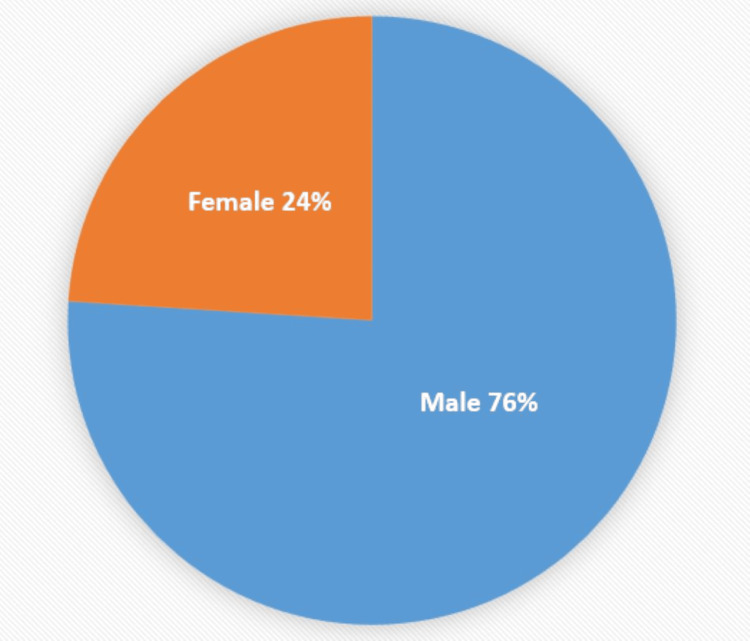
Gender-wise distribution of carbapenem-resistant Klebsiella pneumoniae isolates

The study was carried out on 50 CRKP isolates obtained from clinical samples processed in our laboratory. In the current study, the male: female ratio of the patients from whom CRKP isolates were obtained was found to be 38:12 (76%:24%), respectively, as depicted in Figure 2.

Most of the CRKP isolates were reported among the age group of 51-60 years, accounting for 13 (26%) patients. The least number of isolates were from patients aged 11-20 and 71-80 years, accounting for one (2%) each, respectively. Among 50 CRKP isolates collected, the maximum was isolated from urine samples (19, 38%), followed by pus (15, 30%), endotracheal aspirate (seven, 14%), blood (four, 8%), bronchoalveolar lavage (three, 6%), and sputum (two, 4%). The sample-wise split-up is enlisted in Table 2.

**Table 2:** Sample-wise distribution of carbapenem-resistant Klebsiella pneumoniae isolates

Sample	Number of isolates	Isolates in percentage
Urine	19	38%
Pus	15	30%
Endotracheal aspirate	7	14%
Blood	4	8%
Broncho alveolar lavage	3	6%
Sputum	2	4%

Antibiotic susceptibility pattern of the isolates by the VITEK-2 Compact system

**Figure 3:**
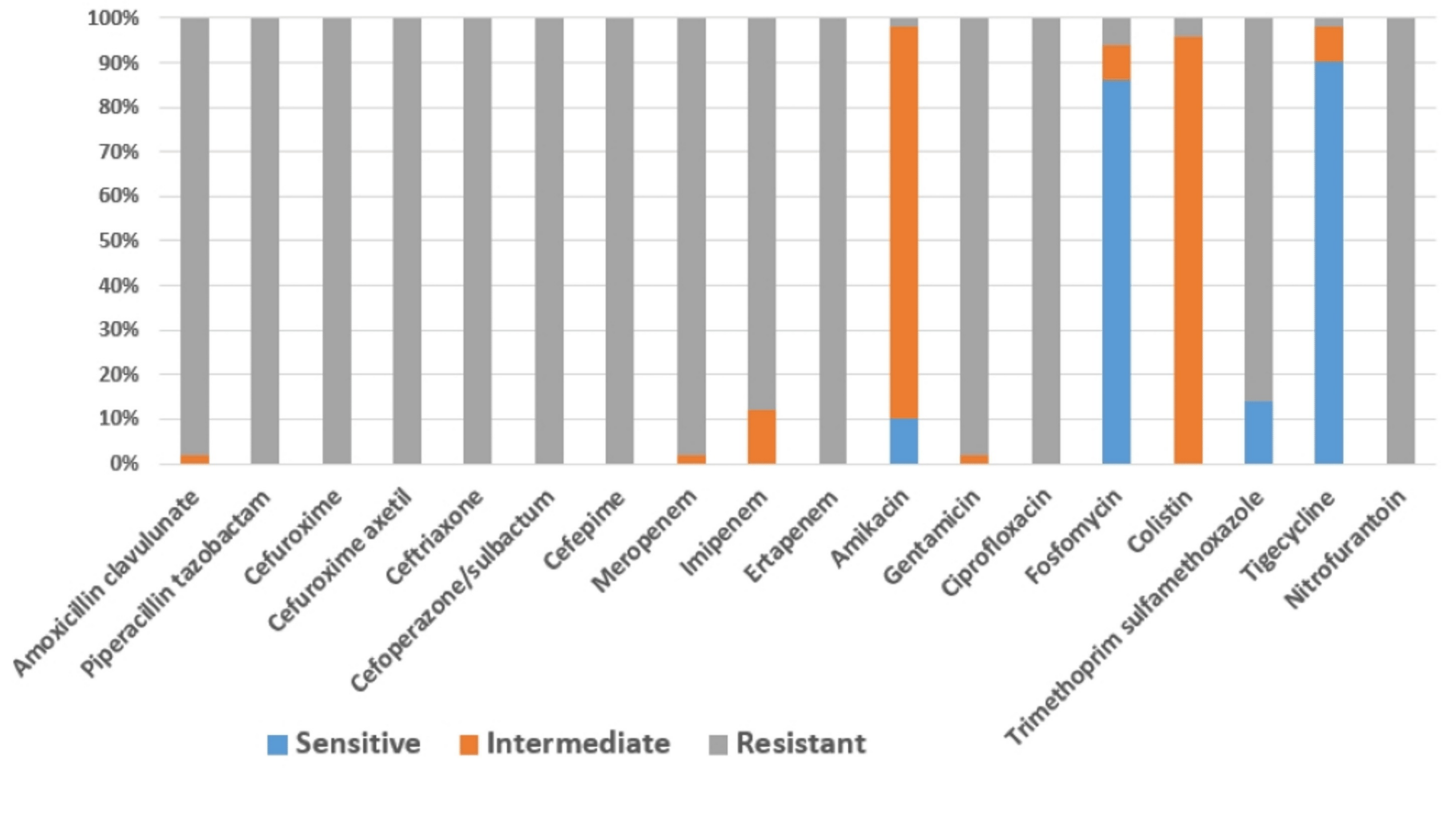
Antibiotic susceptibility pattern of the isolates by the VITEK-2 Compact system

Antibiotic susceptibility analysis revealed resistance of all 50 CRKP isolates to piperacillin-tazobactam, cefuroxime, cefuroxime-axetil, ceftriaxone, cefoperazone sulbactam, cefipime, ertapenem, and ciprofloxacin. Among the isolates included in the present study, only a single CRKP isolate from a urine sample was found to be intermediate to amoxicillin clavulanate. The maximum number of isolates (45, 90%) were sensitive to tigecycline. All the 19 CRKP urine isolates studied were found to be resistant to nitrofurantoin, as depicted in Figure 3.

Minimum inhibitory concentration determination of fosfomycin by the BMD method and VITEK-2 Compact system

Of 50 CRKP isolates, 43 (86%) were sensitive and seven (14%) were resistant to fosfomycin by the VITEK-2 Compact system while 34 (68%) were sensitive, and 16 (32%) were resistant to fosfomycin by the BMD method. Considering BMD as the gold standard, susceptibility obtained by the VITEK-2 Compact system showed an error percentage of 18%.

Minimum inhibitory concentration determination of colistin by the BMD method and VITEK-2 Compact system

Among 50 CRKP isolates, 48 (96%) were intermediate and two (4%) were resistant to colistin by the VITEK-2 system. The BMD testing of colistin revealed that 27 (54%) were sensitive and 23 (46%) were resistant. As per standard guidelines (EUCAST guidelines 2023 and CLSI guidelines 2022), the approved tests for detection of colistin resistance are colistin BMD, colistin agar test, and colistin broth disc elution test. Taking the results of the colistin BMD test as the gold standard, the susceptibility obtained by the VITEK-2 Compact system had an error percentage of 42%.

Synergy testing by BMD-based checkerboard method

Thirty-six (72%) isolates showed synergism, eight (16%) isolates showed indifference, and six (12%) isolates showed partial synergism, while none of them showed additivity and antagonism. These results are found to be statistically significant (chi-square value of 116.204 and p-value of <0.00001) and are depicted in Table 3.

**Table 3:** Synergy testing by the Checkerboard method

Activity	Number of isolates (%)
Synergism	36 (72%)
Partial synergism	6 (12%)
Additivity	Nil
Indifference	8 (16%)
Antagonism	NIL

## Discussion

The magnitude of raising “multidrug-resistant” (MDR) bacteria has created the demand to re-evaluate novel antibiotic choices for treating these infections. Carbapenemase-producing *Klebsiella pneumoniae* is a growing issue among MDR bacteria and is connected to major nosocomial infections [4]. Additionally, concerns are also expressed over a potential rise in community-acquired diseases [8].

A study conducted by Hu et al. has reported a higher rate of CRKP among male patients (73.33%) compared to females (26.67%), which was consistent with our study findings of higher CRKP isolation (38, 76%) among male subjects [19]. In the present study, the highest number of CRKP were isolated from patients aged between 51 and 60 years (13, 26%). Yao et al. have reported the highest number of CRKP isolates among patients aged 18-87 years (62.5%) [20]. In our study, the urine samples had the highest contribution to the total CRKP isolates (19, 38%), which was similar to that of the study by Sung et al., where the highest contribution to the total CRKP isolates was from urine samples (41.7%) [21].

The AST pattern of CRKP isolates showed high resistance to most of the antimicrobial agents tested, which include β- lactam/ β- lactamase inhibitors, cephalosporins, carbapenems, and fluoroquinolones, which is similar to the study findings by Hu et al. and Sung et al. [19, 21].

Fosfomycin is an intracellular phosphoenolpyruvate (PEP) analog that shows an excellent spectrum of activity against both gram-positive and gram-negative bacteria. It is a bactericidal antibiotic that inhibits cell wall synthesis by preventing the formation of peptidoglycan chains [12,22]. A study by Ribeiro et al. revealed that most CRKP isolates were susceptible to fosfomycin (82.3%), and this finding seemed to be consistent with our study, where most of the CRKP isolates were found to be susceptible by the VITEK-2 Compact system (43, 86%) [23]. A study conducted by Bakthavatchalam et al. showed that 52% of CRKP isolates were sensitive to fosfomycin by the agar dilution method, but in our study we have reported 34 (68%) isolates to be sensitive to fosfomycin by the BMD method [10].

A cationic peptide called colistin targets the lipopolysaccharides (LPS) of gram-negative bacteria. Colistin ruptures cell membranes and allows leakage of intracellular components by competitively displacing divalent cations like calcium (Ca2+) and magnesium (Mg2+) from the phosphate groups of membrane lipids [24, 25]. Liu et al. have reported that 93.1% of CRKP isolates were susceptible to colistin, which correlates with our study, where 48 (96%) of the isolates were found to be intermediate for colistin by the VITEK-2 Compact system [5]. In our study, 27 (54%) of CRKP isolates were found sensitive to colistin by the BMD method, as in the study by Bakthavatchalam et al., which showed that 70% of their isolates were sensitive to colistin by BMD [10].

*Klebsiella pneumoniae* and the majority of other drug-resistant bacteria have been documented to be resistant to antibiotic monotherapy, encouraging the adoption of combination treatments as an alternate antibacterial strategy. Treatments for “multidrug-resistant” and extremely drug-resistant gram-negative infections have drawn attention to fosfomycin and colistin [10]. In the current study, synergistic activity between fosfomycin and colistin was seen in 36 (72%) of the CRKP isolates, and eight (16%) of them showed Indifference by the BMD-based Checkerboard method. A study conducted by Chukamnerd et al. has reported 23.8% synergism and 76.2% indifference by BMD-based Checkerboard assay among CRKP isolates [11]. The results obtained by the in vitro BMD-based Checkerboard assay for synergy testing between fosfomycin and colistin are found to be statistically significant (chi-square value of 116.204 and p-value of <0.00001). Similarly, studies by Ontong et al. have also documented definitive in vitro synergism (72.72%) between fosfomycin and colistin among MDR *Klebsiella pneumoniae* isolates [25]. However, a study by Sengel et al. has reported 41% synergism by agar dilution Checkerboard technique among OXA-48 and NDM-producing *Klebsiella pneumoniae* [22]. In addition to this, a study conducted by Evren et al. has reported antagonism against all the OXA-48 positive-*Klebsiella pneumoniae* isolates by the BMD-based Checkerboard method [4]. To establish clear evidence of in vitro synergism, further large-scale studies are needed to investigate the interactions between the pharmacokinetic and pharmacodynamic properties of the drugs.

Limitations of the study

Small Sample Size

The study involved 50 CRKP isolates obtained through different patient samples. These data may not represent the entire spectrum of CRKP strains circulating in the hospital or community, limiting the applicability of the findings.

Single-Center Study

The research was conducted in a single tertiary care hospital located in Mysuru, South India. Regional variations in patient demographics and antimicrobial resistance patterns of CRKP isolates may affect the applicability of the results to other geographic locations.

In Vitro Nature of Synergy Testing

In this study, the Checkerboard method assessed synergism between colistin and fosfomycin in vitro. In vitro results may not always translate directly to clinical outcomes, where factors like bacterial load, tissue penetration of antimicrobial drugs, and pharmacokinetics could influence treatment efficacy differently.

Ethical Considerations

While institutional ethical clearance was obtained, the study's scope was only limited to laboratory-based investigations. Clinical outcomes and patient responses to colistin-fosfomycin combination treatment were not evaluated, which is crucial for assessing the real-world effectiveness of antibiotic combinations.

## Conclusions

This study highlights the present-day scenario of dwindling antibiotic reserves and the increasing resistance among the high-priority CRKP pathogens. Colistin, being a reserve antibiotic, needs careful clinical utilization to deliver maximum benefit to patients. An in vitro combination of fosfomycin-colistin has shown a promisingly significant synergy by Checkerboard BMD testing protocol. Although in vitro testing is one step closer to the evaluation of antimicrobial efficiency, further large-scale studies in this accord encompassing in vivo efficiency would give a clear picture of the holistic effectiveness of this novel combination.

## References

[1] Dai P, Hu D (2022). The making of hypervirulent Klebsiella pneumoniae. J Clin Lab Anal.

[2] Nulsopapon P, Nasomsong W, Pongchaidecha M, Changpradub D, Juntanawiwat P, Santimaleeworagun W (2021). The synergistic activity and optimizing doses of tigecycline in combination with aminoglycosides against clinical carbapenem-resistant Klebsiella pneumoniae isolates. Antibiotics (Basel).

[3] Xu L, Sun X, Ma X (2017). Systematic review and meta-analysis of mortality of patients infected with carbapenem-resistant Klebsiella pneumoniae. Ann Clin Microbiol Antimicrob.

[4] Evren E, Azap OK, Çolakoğlu Ş, Arslan H (2013). In vitro activity of fosfomycin in combination with imipenem, meropenem, colistin and tigecycline against OXA 48-positive Klebsiella pneumoniae strains. Diagn Microbiol Infect Dis.

[5] Liu E, Jia P, Li X (2021). In vitro and in vivo effect of antimicrobial agent combinations against carbapenem-resistant Klebsiella pneumoniae with different resistance mechanisms in China. Infect Drug Resist.

[6] Wang J, He JT, Bai Y, Wang R, Cai Y (2018). Synergistic activity of colistin/fosfomycin combination against carbapenemase-producing Klebsiella pneumoniae in an in vitro pharmacokinetic/pharmacodynamic model. Biomed Res Int.

[7] Allander L, Vickberg K, Lagerbäck P, Sandegren L, Tängdén T (2022). Evaluation of in vitro activity of double-carbapenem combinations against KPC-2-, OXA-48-and NDM-producing Escherichia coli and Klebsiella pneumoniae. Antibiotics (Basel).

[8] Fredborg M, Sondergaard TE, Wang M (2017). Synergistic activities of meropenem double and triple combinations against carbapenemase-producing Enterobacteriaceae. Diagn Microbiol Infect Dis.

[9] Ojdana D, Gutowska A, Sacha P, Majewski P, Wieczorek P, Tryniszewska E (2019). Activity of ceftazidime-avibactam alone and in combination with ertapenem, fosfomycin, and tigecycline against carbapenemase-producing Klebsiella pneumoniae. Microb Drug Resist.

[10] Bakthavatchalam YD, Shankar A, Muthuirulandi Sethuvel DP, Asokan K, Kanthan K, Veeraraghavan B (2020). Synergistic activity of fosfomycin-meropenem and fosfomycin-colistin against carbapenem resistant Klebsiella pneumoniae: an in vitro evidence. Future Sci OA.

[11] Chukamnerd A, Pomwised R, Paing Phoo MT, Terbtothakun P, Hortiwakul T, Charoenmak B, Chusri S (2021). In vitro synergistic activity of fosfomycin in combination with other antimicrobial agents against carbapenem-resistant Klebsiella pneumoniae isolated from patients in a hospital in Thailand. J Infect Chemother.

[12] Liu P, Chen S, Wu ZY, Qi M, Li XY, Liu CX (2020). Mechanisms of fosfomycin resistance in clinical isolates of carbapenem-resistant Klebsiella pneumoniae. J Glob Antimicrob Resist.

[13] Kuai J, Zhang Y, Lu B (2023). In vitro synergistic activity of ceftazidime-avibactam in combination with aztreonam or meropenem against clinical Enterobacterales producing Bla(KPC) or Bla(NDM). Infect Drug Resist.

[14] Dhandapani S, Sistla S, Gunalan A, Manoharan M, Sugumar M, Sastry AS (2021). In-vitro synergistic activity of colistin and meropenem against clinical isolates of carbapenem resistant E.coli and Klebsiella pneumoniae by checkerboard method. Indian J Med Microbiol.

[15] Maryam L, Khalid S, Ali A, Khan AU (2019). Synergistic effect of doripenem in combination with cefoxitin and tetracycline in inhibiting NDM-1 producing bacteria. Future Microbiol.

[16] Hsieh MH, Yu CM, Yu VL, Chow JW (1993). Synergy assessed by checkerboard. A critical analysis. Diagn Microbiol Infect Dis.

[17] Bonapace CR, Bosso JA, Friedrich LV, White RL (2002). Comparison of methods of interpretation of checkerboard synergy testing. Diagn Microbiol Infect Dis.

[18] Laishram S, Pragasam AK, Bakthavatchalam YD, Veeraraghavan B (2017). An update on technical, interpretative and clinical relevance of antimicrobial synergy testing methodologies. Indian J Med Microbiol.

[19] Hu F, Lin ML, Mou JL (2023). Molecular and clinical characteristics of carbapenem-resistant Klebsiella pneumoniae isolates at a tertiary hospital in Wuhan, China. Infect Drug Resist.

[20] Yao H, Liu J, Jiang X, Chen F, Lu X, Zhang J (2021). Analysis of the clinical effect of combined drug susceptibility to guide medication for carbapenem-resistant Klebsiella pneumoniae patients based on the kirby-bauer disk diffusion method. Infect Drug Resist.

[21] Sung CL, Hung WC, Lu PL, Lin L, Wang LC, Yang TY, Tseng SP (2021). Synergistic combination of AS101 and azidothymidine against clinical isolates of carbapenem-resistant Klebsiella pneumoniae. Pathogens.

[22] Erturk Sengel B, Altinkanat Gelmez G, Soyletir G, Korten V (2020). In vitro synergistic activity of fosfomycin in combination with meropenem, amikacin and colistin against OXA-48 and/or NDM-producing Klebsiella pneumoniae. J Chemother.

[23] Ribeiro AC, Chikhani YC, Valiatti TB (2023). In vitro and in vivo synergism of fosfomycin in combination with meropenem or polymyxin B against KPC-2-producing Klebsiella pneumoniae clinical isolates. Antibiotics (Basel).

[24] Geladari A, Simitsopoulou M, Antachopoulos C, Roilides E (2019). Dose-dependent synergistic interactions of colistin with rifampin, meropenem, and tigecycline against carbapenem-resistant Klebsiella pneumoniae biofilms. Antimicrob Agents Chemother.

[25] Ontong JC, Ozioma NF, Voravuthikunchai SP, Chusri S (2021). Synergistic antibacterial effects of colistin in combination with aminoglycoside, carbapenems, cephalosporins, fluoroquinolones, tetracyclines, fosfomycin, and piperacillin on multidrug resistant Klebsiella pneumoniae isolates. PLoS One.

